# Using Balanced Time Perspective to Explain Well-Being and Planning in Retirement

**DOI:** 10.3389/fpsyg.2017.01781

**Published:** 2017-10-13

**Authors:** Anna Mooney, Joanne K. Earl, Carl H. Mooney, Hazel Bateman

**Affiliations:** ^1^Flinders Business School, Flinders University, Adelaide, SA, Australia; ^2^School of Computer Science, Engineering and Mathematics, Flinders University, Adelaide, SA, Australia; ^3^School of Risk and Actuarial Studies, University of New South Wales, Sydney, NSW, Australia

**Keywords:** time perspective, balanced time perspective, planning behavior, well-being, retirement

## Abstract

The notion of whether people focus on the past, present or future, and how it shapes their behavior is known as Time Perspective. Fundamental to the work of two of its earliest proponents, Zimbardo and Boyd (2008), was the concept of balanced time perspective and its relationship to wellness. A person with balanced time perspective can be expected to have a flexible temporal focus of mostly positive orientations (past-positive, present-hedonistic, and future) and much less negative orientations (past-negative and present-fatalistic). This study measured deviation from balanced time perspective (DBTP: Zhang et al., 2013) in a sample of 243 mature adults aged 45 to 91 years and explored relationships to Retirement Planning, Depression, Anxiety, Stress, Positive Mood, and Negative Mood. Results indicate that DBTP accounts for unexplained variance in the outcome measures even after controlling for demographic variables. DBTP was negatively related to Retirement Planning and Positive Mood and positively related to Depression, Anxiety, Stress, and Negative Mood. Theoretical and practical implications regarding balanced time perspective are discussed.

## Introduction

Lewin (1951) originally proposed that an individual's behavior, mood, and morale depends on their psychological view of the past and future “existing at a given time” (p. 75). Extending this idea, Zimbardo and Boyd (1999) developed a conceptual model of time perspective (TP), which includes five temporal categories that have cognitive, affective, and motivational consequences. The five individual TPs proposed were: *Past-Positive*—a tendency to reflect on past events with a sentimental attitude; *Past-Negative*—a focus on the recollection of negative experiences, including the negative reconstruction of mundane events; *Present-Hedonism*—attraction toward immediate pleasure, including risk-taking and sensation seeking, with little concern for future consequences; *Present-Fatalism*—a belief that the future is predestined and humans have no influence on future outcomes; and, *Future*—forward thinking, and goal- setting, while often neglecting present enjoyment. Zimbardo and Boyd (1999) defined these temporal orientations as the manner by which experienced events are encoded, stored, and recalled, as well as the manner in which they form “expectations, goals, contingencies, and imaginative scenarios” (p. 1271).

According to TP theory, cognitive processes shaped by temporal biases define our view of the world and our relationship within it (Keough et al., 1999). Further, every individual has a unique combination of time orientations, with one of the perspectives often dominating more than the others do. However, when individuals are strongly biased toward any one of the five perspectives, their behavioral responses appear to form part of their “personality” and become predictable (Gutpa et al., 2012). By extension, when a particular temporal bias dominates at the expense of other time perspectives, they may become dysfunctional (Boniwell and Zimbardo, 2004) due to an overemphasis on the past, present, or future. For example, strong present focus has been linked to substance abuse (Keough et al., 1999), and risky driving (Zimbardo et al., 1997); while high emphasis on past negative related to personality problems and depression (van Beek et al., 2010); and future focus more likely stimulates academic achievement (Horstmanshof and Zimitat, 2007) and retirement planning (Earl et al., 2015). Similar to personality traits, the theoretical construct of time perspective can be viewed as a single facet of one's individuality, given its widely acknowledged influence on behavior (Gutpa et al., 2012). An important point of difference between TPs and personality, as Zimbardo and Boyd (2008) asserted, is that TPs are not inflexible, and most people can change their temporal orientation if they are motivated to do so and are shown how.

### Balanced time perspective

Balanced time perspective (BTP) was originally proposed as a theoretical concept (Boniwell and Zimbardo, 2004). The idea of temporal balance as a predictor of various well-being measures, and its influence on positive outcomes, has gained research momentum (e.g., see Sobol-Kwapinska and Jankowski, 2016; Stolarski et al., 2016). Zimbardo and Boyd (1999) described BTP as having the mental ability to switch flexibly between temporal orientations as appropriate to meet the demands of any given situation, rather than having a bias toward—or neglect of—a specific TP that may influence important decisions and actions in a maladaptive manner. Zimbardo and Boyd (2008) proposed that in order to maximize well-being and have good psychological health, it was necessary for people to have a temporal balance. They suggested fairly specific parameters required to achieve “balance,” and proposed a “starting point” for operationalizing BTP. Based on the 56-item Zimbardo Time Perspective Inventory (ZTPI; Zimbardo and Boyd, 1999), using a 1–5 scoring scale, a balanced profile as presented on Zimbardo and Boyd's (2016) webpage: www.thetimeparadox.com/surveys/, consists of a combination of high level of present-hedonistic orientation (4.33), moderately high level of past-positive (3.67) and future orientations (3.69), and low levels of past-negative (2.1) and present-fatalistic (1.67) scores. Zimbardo and Boyd (1999) are not explicit about the algorithm used to determine the optimal scores on each of the Time Perspective sub-scales. Whilst it appears that these are theoretical concepts subsequent studies have operationalized the concept and found evidence of links to well-being. An explanation about how these sub-scales scores are used to determine balance is provided in section Deviation from Balanced Time Perspective (DBTP). Specifically, Zimbardo and Boyd have suggested that since TPs are malleable, individuals should work toward achieving a temporal balance for optimal functioning and well-being (Stolarski et al., 2015), rather than being restricted to any particular temporal bias that minimizes the others (Boniwell, 2005).

To date, different methods for measuring balance have been applied (Zhang et al., 2013). For example, Drake et al. (2008), used a cut-off method, Boniwell et al. (2010), used a hierarchical cluster analysis, while Stolarski et al. (2011) used a deviation from BTP (DBTP) method. Despite method used, findings have consistently linked BTP to various well-being indicators. The first two of the three studies mentioned, categorized subjects between “balanced” and not balanced' and, collectively, reported 5–23% of their samples were balanced. Stolarski et al. (2011) on the other hand, adopted a continuous approach. Recognizing the disparity in how BTP was being measured, Zhang et al. (2013) recruited four samples (*N* = 1,739) and assessed the three operationalizations previously used to measure the construct. Of the three methods tested, they reported the best predictor of subjective well-being was the DBTP. That is, rather than measure balance—which categorizes individuals—their study demonstrated that measuring *deviation* from BTP was (a) the best predictor of subjective well-being, and (b) made more sense to measure how *ill-balanced* one was rather than how balanced.

### The influence of balanced time perspective on well-being

TP has been linked to subjective well-being (Drake et al., 2008; Webster et al., 2014); mindfulness (Stolarski et al., 2016); self-esteem, life satisfaction, and optimism (Sobol-Kwapinska and Jankowski, 2016); and positive functioning (Drake et al., 2008; Boniwell et al., 2010). DBTP has also been applied to usefully measure these differences (Zhang et al., 2013). Stolarski et al. (2014) reported evidence of a relationship between DBTP and mood, where low DBTP (closer to optimal) scoring participants experienced more positive mood states and lower tension.

In a separate series of studies testing DBTP's mediating role between mindfulness and life satisfaction, Stolarski et al. (2016) reported DBTP provided a greater unique contribution of the variance in life satisfaction (up to 27%) over mindfulness in all studies. A similar finding identified the mediating role of BTP between temperament and PTSD symptom severity in survivors of motor vehicle accidents (Stolarski and Cyniak-Cieciura, 2016) where the more balanced individuals revealed lower symptom severity. Other studies have investigated ways of addressing balance by managing extreme scores. In their clinical study, Sword et al. (2015) encouraged a focus on the opposite temporal orientation of extreme, or dominant, TPs to create better balance. They treated post-traumatic stress disorder symptoms in serving military personnel and war veterans by targeting maladaptive negative orientations with their opposite positive orientation, and reported significant improvement.

### The influence of balanced time perspective on retirement planning

Throughout this article, retirement planning refers to planning behavior during retirement. Research supports the importance of ongoing planning because retirement is not an event, but rather a process (Kim and Moen, 2002; Donaldson et al., 2010), and setting goals during retirement is fundamental to maintenance of resources (Ebner et al., 2006), and adjustment and well-being (Petkoska and Earl, 2009). Earl et al. (2015) found TP predicted planning preretirement and influenced retirement adjustment and outcomes. The evidence provided by Earl et al.'s 18-month longitudinal study suggests that TP orientations influence the propensity to plan. Extending on Earl et al.'s research, the present study investigated whether variances in retirement planning behavior can be explained by DBTP.

### Study aims and hypotheses

In light of the reported evidence linking temporal balance with well-being indicators, the current study aimed to progress our understanding of BTP in older individuals by: (a) testing the relationship between DBTP and planning behavior, and (b) exploring the association between DBTP and well-being. Consistent with Drake et al. (2008) and Boniwell et al. (2010), we expected that between 5 and 23% of the sample would be defined as balanced (H1). This approach was applied to assess whether the current, older, sample was different in terms of proportion of balanced participants, to much younger samples in the literature. We also predicted that greater DBTP would be negatively related with planning and positive affect (H2), and positively related with depression, anxiety, stress, and negative affect (H3). We used both the original BTP profile, as advocated by Zimbardo and Boyd (2008), as well as their recently updated balanced profile taken from their webpage: www.thetimeparadox.com/surveys/. We hypothesized that DBTP will account for additional unique variance in all outcome variables after controlling for demographic information (H4).

## Method

### Participants and procedure

One hundred and twenty-seven self-nominated retirees were recruited, primarily through My Longevity, a web-based provider of information about age-related risk factors and tips on boosting life expectancy. Participants were also sought from the general public through invitation pamphlets distributed at various council community centers. The age range of participants was 52–91 years. Men represented 66.9% of the sample (*n* = 85). Fully retired individuals made up 62.2% (*n* = 79), and 37.8% (*n* = 48) were partially retired. Relationship status was reported as 65.4% (*n* = 83) married, 14.2% (*n* = 18) de facto relationship, 5.5% (*n* = 7) widowed, 11.8% (*n* = 15) divorced or separated, and 3.1% (*n* = 4) were single. In terms of educational achievement, 11.8% (*n* = 15) reported they had not completed high school, 6.3% (*n* = 8) completed high school, 7.9% (*n* = 10) graduate diploma, 10.2% (*n* = 13) advanced diploma, 25.2% (*n* = 32) Bachelor's degree, 37% (*n* = 47) post-graduate degree, and 1.6% (*n* = 2) reported “other.”

Individuals were invited to participate in the study via email and those interested responded by emailing the researcher. A link to the survey was then emailed to interested respondents, who first read the participant information statement before consenting to proceed with participation.

### Ethical considerations

In line with the National Statement on Ethical Conduct in Human Research (Australian Government, 2007), ethical concerns were addressed prior to ethics approval, and throughout the research process. Respondents were provided with information about the study and they acknowledged the absence of a guarantee of any benefits or improvements to them because of their participation. Participants were assured of confidentiality of identifiable information and were informed of their right to withdraw participation at any time without prejudice from any party. No incentives were offered.

### Materials

We used an online survey design to collect information from participants. Existing, reliable measures and previously published measures were used. Chronbach's alpha reliability coefficients from original studies are shown, while alphas from the current study are reported in Table 1.

**Table 1:** Bivariate Correlations with Means, Standard Deviations, and Cronbach's Alpha Reliability Coefficients (where applicable).

**Variables**	***M***	***SD***	**α**	**1**	**2**	**3**	**4**	***5***	**6**	**7**
1. Age	68.65	6.96								
2. RPQII	83.50	18.15	0.86	0.03						
3. Depression	19.*o*4	6.76	0.86	−0.03	−0.26^*^					
4. Anxiety	16.02	3.22	0.69	0.01	0.10	0.41^**^				
5. Stress	21.13	6.86	0.86	0.02	0.08	0.46^**^	0.53^**^			
6. Positive affect	17.76	3.82	0.87	−0.08	0.20^*^	−0.62^**^	−0.46^**^	−0.41^**^		
7. Negative affect	8.74	4.42	0.90	0.09	−0.16	0.54^**^	0.41^**^	0.49^**^	−0.60^**^	
8. DBTPb	1.86	0.61		−0.06	−0.34^**^	0.51^**^	0.36^**^	0.31^**^	−0.48^**^	0.43^**^

*N = 127*.

*^a^ Retirement Planning Questiormaire (RPQII; Muratore and Earl, 2010)*.

*^b^ Deviation from Balanced Time Perspective*.

* *p < 0.05.*,

** *p < 0.001*.

#### Zimbardo time perspective inventory (ZTPI: Zimbardo and Boyd, 1999)

The 56-item ZTPI is a self-report measure consisting of five subscales designed to identify an individual's TP: *Past-Negative* (α = 0.82); *Past-Positive* (α = 0.80); *Present-Hedonistic* (α = 0.79); *Present-Fatalistic* (α = 0.74); and *Future* (α = 0.77). Each item is on a five-point Likert scale ranging from 1 (*very uncharacteristic*) to 5 (*very characteristic*). Higher scores reflecting a stronger orientation toward that particular item's TP.

#### Deviation from balanced time perspective (DBTP)

The method for calculating DBTP in this study was that proposed by Zhang et al. (2013). Deviation from balance was calculated by the sum of the distance away from each optimal TP scored, and then squared—commonly referred to as the Squared Euclidean metric. To clarify, a score of zero indicates the perfect balance, therefore, the higher the DBTP score the more *illbalanced* one is. This involved subtracting each empirical (*e*) TP from the ideal (*i*) and squaring it, thus eliminating negative values. The derived values for each of the five TPs were then summed, and the square root was taken (see Equation 1). This method calculated the total distance a participant deviated from zero—the balanced ideal. Calculations were performed using Microsoft Excel (2013).

(1) $$DBTP=\sqrt{\begin{array}{l} {\left(iPN-ePN\right)}^{2}+\;{\left(iPP-ePP\right)}^{2}+{\left(iPF-ePF\right)}^{2}+\;\\ {\left(iPH-ePH\right)}^{2}+\;{\left(iF-eF\right)}^{2} \end{array}}$$

#### Retirement planning questionnaire (RPQII; Muratore and Earl, 2010)

The 28-item RPQII was used to measure the amount of effort participants invested in retirement planning. The measure was developed to assess a range of behaviors relevant to retirement including accessing services offered through government agencies, financial, health, social and other non-financial planning. Internal reliability in original study was α = 0.86. Participants were assessed on their effort contributed toward retirement planning on a scale of 1 (very small amount of effort) to 5 (very large amount of effort). Scores for each participant were obtained by summing the ratings on all items and averaging; higher scores indicated greater effort invested on retirement planning.

#### Mood questionnaire (Efklides and Petkaki, 2005)

The 10-item measure assesses state mood. Participants rated the level of applicability of each adjective in describing the way they feel using a 5-point scale from (1) *Least Applicable* to (5) *Most Applicable*. Examples of items include “*Happy,” “Excited,”* and “*Anxious.”* Scores were calculated separately for positive mood and negative mood, ranging from 5 to 25. Higher scores reflected stronger positive or negative mood. The original study had good internal reliability for both positive (α = 0.70) and negative (α = 0.81) measures (Field, 2013).

#### Depression anxiety stress scale-21 (Lovibond and Lovibond, 1995)

The DASS-21 is a psychometric tool that has been widely administered, especially in clinical settings. It is a shorter version of the original DASS, and its psychometric properties were established in a study by Antony et al. (1998). Participants rated the applicability of each statement to themselves on a 4-point Likert scale with anchor points from (0) “*did not apply to me at all”* to (3) “*applied to me very much, or most of the time*.” Ratings within each subscale were summed, and then multiplied by 2, in order to obtain scores for each subscale. Possible scores range from 0 to 34, depending on the number of items each subscale has. Higher scores indicated greater level of stress, anxiety or depression.

## Results

The data collected were screened for assumptions of normality, and missing data, before conducting any analysis.

### Proportion of balanced participants

We report the results of sub-scale TP scores from our sample to compare with the normative sample from Zimbardo and Boyd (2016) on which the balanced profile was based.

The sample means from this study were; past-negative 2.44, past-positive 3.89, present-fatalism 2.15, present hedonism 3.11, and future 3.76. Normative sample means reported by Zimbardo and Boyd (2016) were past-negative 3.00, past-positive 3.22, present-fatalism 2.33, present hedonism 3.93, and future 3.38. The ideal TP scores recommended by Zimbardo and Boyd (2016) to calculate balance are as follows: past-negative, 2.10; past-positive, 3.67; present-fatalism, 1.67; present-hedonism, 4.33; and future, 3.69.

Our focus was on the balanced profile from which the TP subscale scores were based; we used only DBTP scores. As reported by Zhang et al. (2013), DBTP was the best predictor of subjective well-being. Thus, in the current study, level of balance was determined by how much scores deviated from zero (the balanced ideal) on the DBTP continuum.

In the current study, for the purpose of comparing our sample with other samples in the literature, well-balanced individuals were identified as having a DBTP score ≥ 1 *SD* below the mean; that is, a low level of DBTP (more balance) was indicated by a score of ≤ 1.23. In accord with previous studies reporting 5–23% of participants were balanced (Drake et al., 2008; Boniwell et al., 2010), the proportion of participants with low level of DBTP was 14.2% (*n* = 18), thus confirming our first hypothesis.

### Relationship between DBTP and outcome measures

Descriptive statistics, Cronbach's alpha (where applicable), and correlation coefficients for all investigated variables, are presented in Table 1. In terms of demographic information, 62.2% (*n* = 79) were fully retired, a large proportion of the sample were married or in a de facto relationship (79.6%, *n* = 101), and a large proportion were educated at tertiary level (72.4%, *n* = 92). All statistical analyses were conducted using the software program, IBM Corp (2013). As shown, there is no significant association between DBTP and age. However, the correlational analysis revealed a significant relationship between DBTP and all of the outcome variables; retirement planning, DASS subscales, and mood.

Therefore, we tested whether DBTP accounted for additional unique variance in retirement planning, depression, anxiety, stress, positive mood, and negative mood by conducting a series of multiple regression analyses. Demographic variables, age, gender, and relationship status (single or in a relationship) were controlled for in all the models by entering them in Step 1. DBTP scores were entered in step 2. As reported in Table 2, the regression analyses mirrored the results of the zero-order correlations and confirmed our hypotheses in terms of the direction and significance of the scores. The regression analyses tested DBTP relationships with the outcome variables. As expected, DBTP was negatively related with retirement planning, *F*_(1, 121)_ = 21.26, *p* < 0.001, and positive affect, *F*_(1, 121)_ = 40.35, *p* < 0.001 (H2). Also, as anticipated, DBTP was positively related with depression, *F*_(1, 121)_ = 46.10, *p* < 0.001, anxiety, *F*_(1, 121)_ = 19.74, *p* < 0.001, stress *F*_(1, 121)_ = 13.69, *p* < 0.001, and negative affect *F*_(1, 121)_ = 31.65, *p* < 0.001 (H3).

**Table 2:** Hierarchical Regression analyses predicting Deviation from Time Perspectives for Retirement Planning^a^, DASS^b^ subscales, and Mood^c^.

	**Hierarchical regression models using balanced time perspective profile**											
	**RPQJib^a^**		**Depression^c^**		**Anxiety^c^**		**Stress^c^**		**Positived^d^**		**Negatived^d^**	
	**Δ*R*^2^**	**β**	**Δ*R*^2^**	**β**	**Δ*R*^2^**	**β**	**Δ*R*^2^**	**β**	**Δ*R*^2^**	**β**	**Δ*R*^2^**	**β**
Step 1	0.05		0.06	0.07^*^		0.01		0.09^*^		0.05		
Demographics^a^												
Step2	0.10^**^		0.23^**^		0.11^**^		0.09^**^		0.20^**^		0.16^**^	
DBTP		−0.32^**^		0.49^**^		0.33^**^		0.31^**^		−0.45^**^		0.40^**^

*N = 127*.

a *Demogra!lhics include age, gender, and relationship status*.

b *Retirement Planning Questiotmaire (RPQII; Muratore and Earl, 2010)*.

c *Subscale affect variables from the Depression Anxiety Stress Scale (DASS-21; Lovibond and Lovibond, 1995)*.

d *Subscale mood variables fi·om Mood Questionnaire (Efklides and Petkaki, 2005)*.

* *p < 0.05*,

** *p < 0.001*.

Other than a small demographic contribution to the variance in the anxiety and positive mood models, there was no demographic contribution to retirement planning, depression, stress, or negative mood. DBTP scores contributed to a significant portion of additional variance in all of the outcome variables. Therefore, our hypothesis regarding additional unique variance in retirement planning, DASS subscales, and positive and negative mood was supported (H4). The evidence strengthens the validity of DBTP as a predictor of planning and subjective well-being.

## Discussion

The aim of the present study was to: (a) investigate consistency in the prevalence of BTP, as defined by Zimbardo and Boyd (2016), and (b) investigate relationships between BTP, retirement planning and well-being measures in individuals who have either fully or partially retired. Our findings reveal that older individuals who deviate less from the balanced ideal are happier, report feeling less stressed, less depressed, less anxious, and they are in a more positive mood. They also care about their future more because they actively make more plans than those who are less balanced.

Our hypotheses regarding the relationship between DBTP and all of the outcome variables were confirmed. That is, DBTP explained additional unique variance in retirement planning, depression, anxiety, stress, positive and negative mood, beyond demographic characteristics. As a result, DBTP was significantly related to planning and well-being. Similar to the prevalence of balanced individuals reported in previous studies, 14.2% met the balanced profile criteria.

### Balanced time perspective and planning behavior

In the present study, DBTP explained an additional contribution to the variance in planning. The results revealed participants with a more balanced temporal view were more likely to be proactive in making plans toward their retirement. Every item on the RPQII (Muratore and Earl, 2010) planning measure asked respondents about what they have *actioned* in terms of planning behavior, rather than what they *plan* to do. Thus, we could reliably conclude that having a healthy time balance allows one the ability to alternate between different temporal views of their life, as necessary, in order to put measures in place to ensure they promote or maintain their well-being during their retirement (Muratore and Earl, 2015). For example, since planning for the future is underpinned by the aspect of time (Zimbardo and Boyd, 1999), balanced people are better able to make relevant future plans that are informed by an evaluation of past experiences and their current lifestyle rather than, say, considering just the preservation of their present lifestyle. Although the current findings link a balanced profile to retirement planning, in the absence of data at a second time point, we cannot conclude causation with certainty. Whether good retirement planning is a result of having temporal balance, or vice versa, could be determined in future research.

### Balanced time perspective and well-being

Zimbardo and Boyd's (1999)notion of a BTP emphasizes the importance of having the ability to switch between temporal orientations, as an adaptive or coping mechanism, through life's demanding experiences. A balanced individual is assumed to possess temporal harmony (Stolarski et al., 2015), and one who functions optimally within a flexible set of all temporal frames depending on the demands of a situation, their values, and needs (Zimbardo and Boyd, 2008).

Consistent with findings of other studies investigating relationships between time perspective and life satisfaction (e.g., Drake et al., 2008; Boniwell et al., 2010; Zhang and Howell, 2011), participants with lower DBTP scores had higher scores on positive variables (e.g., positive affect), and lower scores on negative variables (e.g., depression), compared to those with higher deviations from the optimal profile. Zhang et al. (2013) reported significant relationships between BTP and various well-being measures, regardless of the method used to measure balance. The findings in the present study are in accord with similar findings in the literature that relate to the link between BTP and well-being. It is important to note that the mean age of subjects used in such previous studies were far younger than that of the current sample. For example, Zhang et al.'s (2013) samples averaged below 33 years (*M*_Sample1_ = 25.69, *M*_Sample2_ = 23.39, *M*_Sample3_ = 24.84, *M*_Sample4_ = 32.99); Stolarski et al. (2011) sample averaged 23.8 years; and Sobol-Kwapinska and Jankowski's (2016) sample averaged 37 years. By extending the age range of participants, the current study provides a small contribution to not only the retirement well-being literature, but also to research of the concept of balanced time perspective.

## Limitations and future recommendations

The current study explored BTP relationships with retirement planning, negative emotional states, and mood. A limitation of the current study is that the sample was primarily sourced through My Longevity, and as members of a retirement organization associated with living well, participants may have already been conscientious planners. Future research should source participants more widely to ensure greater diversity in the participant pool and increase generalizability. It may also be worth investigating differences in BTP between recent vs. older retirees. In such a study, DBTP scores between groups may be expected to vary due to differences in individuals' projection into the future. Older retirees, say, 70 years or older, may be more inclined to reconcile with circumstances of the past and think more about enjoying the present; looking after their health and retaining their independence. In contrast, younger retirees may look to the future in preparation for an anticipated long retirement experience.

The current sample comprised of self-nominated retirees. Whether they were fully disengaged from, or still engaged in, paid employment, they were “psychologically” retired. Future research could compare working self-nominated retirees with other workers to investigate the reasons for their continued engagement with work. Are they working for financial reasons, or for enjoyment? Their reasons for working could have implications for their retirement and well-being. Furthermore, their reasons for why they work may make a difference to other variables.

The theory behind BTP posits that a deviation from each individual time domain in either a positive or a negative direction is taken as a deviation from the ideal. This logic presents two issues: (1) excessive deviations from negative orientations are assessed the same way as excessive deviations from positive orientations, despite affective differences; and (2) the contribution of time perspectives is not even in this model, with only one future time perspective compared to two past, and two present perspectives. As a way to further validate DBTP, future research should consider investigating the negative effects of scoring extremely high on positive dimensions, or extremely low on negative dimensions. Despite the positive face value of these extremes, it would be worth investigating whether deviations in these directions hold true to the assumptions of poor functioning associated with ill-balance.

As mentioned, previous studies have advanced the theoretical concept of BTP and operationalized it in ways that categorize individuals as either “balanced” or “not balanced” due to their methodological approaches. A different methodological perspective, deviation from the balanced ideal (Zhang et al., 2013), maintains a continuous approach; the approach we have taken. There is still some conjecture about whether deviations vary depending on what the individual TPs are that deviate, or whether there are, in fact, different balanced types that vary in levels of functioning. Despite the consistent findings linking balance to various well-being measures, the question of whether BTP should be a continuous, or categorical, variable is not yet determined with reasonable confidence. Given the issue of cognitive decline in older age, and taking into account the evidence to date supporting the benefits of balance across numerous domains, future research into how balance affects well-being variables in older cohorts is definitely worth further exploration.

## Ethics statement

This study was conducted in line with the ethical standards as outlined by the National Statement on Ethical Conduct in Human Research. Ethics approval for the original data set was provided by the UNSW Human Research Ethics Advisory Panel C (Psychology) - application number 2487.

## Author contributions

AM completed the study as part of an Honours in Psychology submission. JE supervised this project and secured funding. CM assisted with the development of the formula and application of the deviation from balance time perspective to create the datafile. HB helped secure original funding and reviewed manuscripts.

### Conflict of interest statement

The authors declare that the research was conducted in the absence of any commercial or financial relationships that could be construed as a potential conflict of interest.
